# Using isotope pool dilution to understand how organic carbon additions affect N_2_O consumption in diverse soils

**DOI:** 10.1111/gcb.16190

**Published:** 2022-05-04

**Authors:** Emily R. Stuchiner, Joseph C. von Fischer

**Affiliations:** ^1^ Graduate Degree Program in Ecology Colorado State University Fort Collins Colorado USA; ^2^ Department of Biology Colorado State University Fort Collins Colorado USA

**Keywords:** ^15^N isotope pool dilution, microbial N‐cycling genes, nosZ gene abundance, organic carbon amendments, soil N_2_O emission, soil properties, stimulate N_2_O consumption

## Abstract

Nitrous oxide (N_2_O) is a formidable greenhouse gas with a warming potential ~300× greater than CO_2_. However, its emissions to the atmosphere have gone largely unchecked because the microbial and environmental controls governing N_2_O emissions have proven difficult to manage. The microbial process N_2_O consumption is the only know biotic pathway to remove N_2_O from soil pores and therefore reduce N_2_O emissions. Consequently, manipulating soils to increase N_2_O consumption by organic carbon (OC) additions has steadily gained interest. However, the response of N_2_O emissions to different OC additions are inconsistent, and it is unclear if lower N_2_O emissions are due to increased consumption, decreased production, or both. Simplified and systematic studies are needed to evaluate the efficacy of different OC additions on N_2_O consumption. We aimed to manipulate N_2_O consumption by amending soils with OC compounds (succinate, acetate, propionate) more directly available to denitrifiers. We hypothesized that N_2_O consumption is OC‐limited and predicted these denitrifier‐targeted additions would lead to enhanced N_2_O consumption and increased nosZ gene abundance. We incubated diverse soils in the laboratory and performed a ^15^N_2_O isotope pool dilution assay to disentangle microbial N_2_O emissions from consumption using laser‐based spectroscopy. We found that amending soils with OC increased gross N_2_O consumption in six of eight soils tested. Furthermore, three of eight soils showed **I**ncreased N_2_O **C**onsumption and **D**ecreased N_2_O **E**missions (ICDE), a phenomenon we introduce in this study as an N_2_O management ideal. All three ICDE soils had low soil OC content, suggesting ICDE is a response to relaxed C‐limitation wherein C additions promote soil anoxia, consequently stimulating the reduction of N_2_O via denitrification. We suggest, generally, OC additions to low OC soils will reduce N_2_O emissions via ICDE. Future studies should prioritize methodical assessment of different, specific, OC‐additions to determine which additions show ICDE in different soils.

## Graphical abstract

Nitrous oxide (N_2_O) is a potent greenhouse gas. The microbial process N_2_O consumption is the only know biotic pathway to remove N_2_O from soil pores and therefore reduce N_2_O emissions. We aimed to stimulate N_2_O consumption by amending eight different soils with organic carbon (OC) compounds more directly available to denitrifiers. OC amendments increased N_2_O consumption in 6/8 soils, and 3/8 soils showed **I**ncreased N_2_O **C**onsumption and **D**ecreased N_2_O **E**missions (ICDE). All ICDE soils had low OC, suggesting ICDE is a response to relaxed C‐limitation. We suggest, generally, OC additions to low OC soils will reduce N_2_O emissions via ICDE.

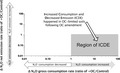

## 1 INTRODUCTION

Nitrous oxide (N_2_O) is a potent greenhouse gas (GHG) that has a 100‐year warming potential 298× greater than carbon dioxide (CO_2_), and it is the primary stratospheric ozone depleting substance (Intergovernmental Panel on Climate Change, 2014; Ravishankara et al., 2009). Over the past 40 years, anthropogenic N_2_O emissions have increased 30%, primarily due to inorganic nitrogen (N) fertilization of croplands (Tian et al., 2020). Unfortunately, it has been challenging to reduce N_2_O emissions by cutting N inputs because N‐fertilizer use is viewed as intrinsic to meeting crop yield demands (Houser & Stuart, 2020; Kanter, Ogle, et al., 2020; Reay et al., 2012; Smith, 2017).

Ideally, the biogeochemical processes that regulate N_2_O emission from croplands could be managed to diminish N_2_O emissions. However, N_2_O management has been challenged by the underlying diversity and complexity of soil N transformations associated with N_2_O production (Butterbach‐Bahl et al., 2013), and by the differing sensitivity of these processes to state factors (Domeignoz‐Horta et al., 2018), transient effects like moisture and temperature (Luo et al., 2013; Schindlbacher et al., 2004), management history (Krause et al., 2017), and details of the N_2_O production intervention (Borchard et al., 2019; Hellman et al., 2019; Lam et al., 2017; Lazcano et al., 2021; Subbarao et al., 2006; Zhou et al., 2017).

The observation of N_2_O uptake from soils, as measured by gas flux chambers (Chapuis‐Lardy et al., 2007) has focused attention on the process of N_2_O consumption in soils, and it highlighted the possibility that N_2_O emissions may be cut by managing the N_2_O consumption process (Box 1, Yoon et al., 2019). The only known biological pathway for N_2_O consumption occurs in denitrifying bacteria where the NosZ enzyme catalyzes the respiratory reduction of N_2_O to N_2_ (Hallin et al., 2018; Richardson et al., 2009). Soil metagenomic studies of the nosZ gene suggest that the N_2_O consuming community is diverse and abundant (Bakken & Frostegård, 2017; Jones et al., 2013, 2014). But despite this conceptual potential for managing N_2_O consumption, Stein (2020) noted in a recent review that significant knowledge gaps prevent us from predicting the response of N_2_O consumption to manipulation or controlling it at the scale of ecosystems.

BOX 1 **Use of isotope pool dilution to quantify rates of gross N_2_O consumption**

To disentangle the kinetics of N_2_O production, consumption, and net emission, we used isotope pool dilution, an assay where isotopically enriched ^15^N_2_O is injected into the headspace of a closed incubation system and then the label ^15^N_2_O disappearance is monitored over time. Soil and atmospheric gases (pools bounded by dotted outlines) are in dynamic equilibrium, maintained by diffusion and gas concentration gradients induced by soil biology over the 48‐h incubation period of the study. Thus, the N_2_O and label ^15^N_2_O concentrations measured in the headspace of the incubation system were ultimately driven by biological processes of N_2_O production and consumption in the soil. Red arrows indicate fluxes that affect the bulk N_2_O pool while blue arrows indicate fluxes that affect the label ^15^N_2_O. In this study, we quantify gross N_2_O consumption by tracking the disappearance of label ^15^N_2_O over time. We also quantify net emission as the change in bulk N_2_O concentration over time.

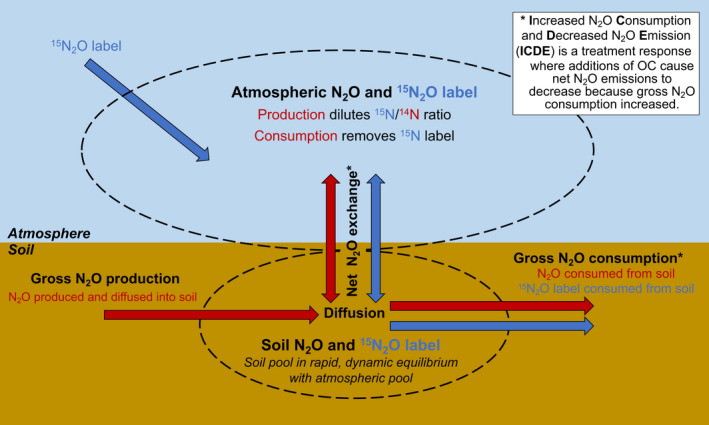

In our experiment, we sought to stimulate N_2_O consumption and thereby reduce net N_2_O emissions. To enhance N_2_O consumption, we added OC to soils and compared the N_2_O consumption rates and net N_2_O emission rates in control versus manipulated soils. In response to OC amendment, we looked for **I**ncreased gross N_2_O **C**onsumption and **D**ecreased net N_2_O **E**missions (ICDE) in soils. From a GHG management perspective, it would be ideal to induce ICDE in any soil. Here we explored how amending soils with organic carbon (OC) provides an electron donor source for enhanced N_2_O → N_2_ reduction, which corresponds to N_2_O consumption. When some soils showed an ICDE response, but others did not, we examined the factors that explained the response.

One major area of uncertainty about stimulating N_2_O consumption is how altering organic matter supply might affect the N_2_O sources and sinks. Organic matter additions have been the focus of many N_2_O management studies (Hellman et al., 2019; Lazcano et al., 2021; Senbayram et al., 2012; Stevenson et al., 2011; Sutton‐Grier et al., 2011; Wang, Amon, et al., 2021; Wu et al., 2018). N_2_O consumption is primarily an anaerobic, respiratory pathway (Hein & Simon, 2019; Shan et al., 2021), and so there exists an environmental correlation where N_2_O consumption rates are highest when both O_2_ concentrations are low and organic matter abundance is high (Conthe et al., 2018; Senbayram et al., 2012; Wu et al., 2013, 2018). However, studies have found that the net emission of N_2_O can go either up or down, depending on the soil and the nature of the organic matter addition. For example, manure additions may create more anoxic conditions, but these additions also provide nitrogen, leading to potential stimulation of N_2_O production (Butterbach‐Bahl & Dannenmann, 2011; Duan et al., 2019). Additionally, sugar additions (e.g., glucose) have most commonly caused increased N_2_O emissions and reduced nosZ abundance (Barrett et al., 2016; Miller et al., 2009; Mitchell et al., 2013; Morley & Baggs, 2010), although they have also contributed to anoxia and a reduction in N_2_O emissions from soil microcosms (Sánchez‐Martín et al., 2008). It is becoming clear that simplified and systematic studies are needed to evaluate the efficacy of different organic carbon (OC) additions on N_2_O consumption.

A second major area of uncertainty arises from the challenge of measuring N_2_O production and consumption (Almaraz et al., 2020; Groffman et al., 2006). When studies find differing N_2_O emission responses to organic matter addition, it is often not clear if the differences arise from changes in N_2_O production, N_2_O consumption or both (Butterbach‐Bahl & Dannenmann, 2011; Conthe et al., 2019; Duan et al., 2019; McMillan et al., 2016; Shan et al., 2021; Wang et al., 2019; Zhu et al., 2015). Ideally, management intervention would induce **I**ncreased N_2_O **C**onsumption and **D**ecreased N_2_O **E**missions (ICDE, Box 1), but other outcomes are, of course, possible.

To shed light in these areas of uncertainty, we addressed the question: does the addition of non‐fermentable organic acids lead to ICDE in diverse soils? Our approach used a ^15^N_2_O isotope pool dilution assay on sieved soils in a controlled laboratory setting. To measure the rates of N_2_O consumption and emission, we used a Los Gatos Research (LGR) laser‐based N_2_O isotopic analyzer. This methodology is distinct from previous studies that used gas flow‐through of intact soil cores (Bourbonnais et al., 2021; Butterbach‐Bahl et al., 2002; Lewicka‐Szczebak et al., 2017; Lewicka‐Szczebak & Well, 2020; Wen et al., 2016) or field‐based isotope pool dilution (Yang et al., 2011). To our knowledge, it is the first laboratory‐based soil incubation using trace gas isotope pool dilution (von Fischer & Hedin, 2002) to measure N_2_O consumption. Our incubation of sieved soils in sealed containers permits a more direct assay of the metabolic potential of soil microbes to consume N_2_O for three reasons. First, sieving removes the inherent physical heterogeneity of intact soils (e.g., soil aggregates, prismatic structures, pore networks) that can limit exchange of the ^15^N_2_O label between the headspace and the loci of consumption. Second, added organic matter can be more homogeneously distributed through sieved soils than intact soils. Third, we depend on a 48‐h incubation time to maximize diffusive exchange of the ^15^N_2_O label between soil microbes and the incubation container headspace. In addition, the laser‐based N_2_O isotopic analyzer is effective at near‐ambient N_2_O concentrations (e.g., does not require a pre‐concentration unit), has much faster throughput (~15 min per sample), and minimal sample preparation (Stuchiner et al., 2020).

Existing alternatives to measure the N_2_O consumption rate (e.g., Helium gas‐flow soil cores and ^15^N gas flow methods; Butterbach‐Bahl et al., 2002; Lewicka‐Szczebak et al., 2017) can quantify N_2_O consumption by measuring both N_2_O and N_2_ production, as calculated from the N_2_O production ratio (i.e., N_2_O /[N_2_ + N_2_O]). However, these methods use gas chromatography (GC) or conventional isotope ratio mass spectrometry (IRMS). IRMS typically requires significantly elevated N_2_O concentrations and/or pre‐concentration (Almaraz et al., 2020; Clough et al., 2006). Moreover, the cost and complexity of purchasing and operating a GC or an IRMS are significantly higher than the laser‐based system that we used, and both IRMS and gas flow‐through systems have relatively slow sample throughput (Ostrom & Ostrom, 2017). In addition, the use of ^15^N‐labelled nitrate (^15^NO_3_^−^) in the ^15^N gas flux method can yield ^15^N_2_O through a wide variety of processes including bacterial denitrification, fungal denitrification, dissimilatory nitrate reduction to ammonium (DNRA), or abiotic processes, etc. (Almaraz et al., 2020; Kulkarni et al., 2014). In contrast, our additions of ^15^N_2_O during isotope pool dilution reduce the metabolic ambiguity by directly quantifying N_2_O consumption through denitrification. We propose that the ability to rapidly and systematically screen the effects of various OC additions on N_2_O consumption rates is needed for improved management of N_2_O consumption. Thus, this approach, using sieved soils and laser‐based analysis of ^15^N_2_O isotope pool dilution in closed‐system assays, could be applied in future studies to achieve this screening.

Based on previous work (Hedin et al., 1998; Ostrom et al., 2002) that stimulated N_2_O consumption through the addition of OC compounds known to be used by denitrifiers (acetate, succinate, propionate), we hypothesized that N_2_O consumption is generally OC limited. In this experiment, we aimed to manipulate N_2_O consumption by amending soils with the abovementioned electron donors that are more exclusively available to denitrifiers, rather than to fermenters. We predicted that these targeted additions would stimulate nosZ abundance and lead to enhanced N_2_O consumption (Kuypers et al., 2018). To test this, we assayed diverse soils collected from Colorado, New Mexico, and Minnesota, (1) to see how amending them with denitrifier‐specific OC compounds would broadly affect N_2_O emission and consumption among soils, and (2) to investigate how underlying properties of the different soils would impact their capacity to consume N_2_O. All soils were held at 60% water saturation, amended with non‐fermentable organic acids (acetate, succinate, propionate), and incubated in the laboratory for 48 hr. We used the above‐described novel ^15^N_2_O isotope pool dilution technique to disentangle microbial N_2_O emissions from consumption (Box 1). We also measured a suite of soil properties and microbial N‐cycling gene abundances to examine which of these factors individually or collectively helped to explain the soil N_2_O dynamics we observed.

## 2 MATERIALS AND METHODS

### 2.1 Field sampling and soil characterization

Soils were collected from eight locations in the United States (Table 1). Five soils were collected in Colorado, one soil was collected in New Mexico, and two soils were collected in Minnesota. Site and soil‐specific properties are summarized in Table 1. All soils were collected within July–August 2019. Throughout the remainder of the article, soils will be referred to by their soil name in Table 1.

**TABLE 1:** Summary of environments where soils were collected, and features of all soils used in the incubation experiment

Site name	Location	Soil name	GPS coordinates	MAT (°C)	MAP (mm)	Soil classification	Soil horizon
Shortgrass Steppe	Nunn, CO	Shortgrass prairie	40.80458, −104.71565	11	381	Alluvial Nunn, fine clayey loam	Ap/Bt
Limited Irrigation Research Farm	Greeley, CO	Colorado cornfield	40.44852, −104.63897	9.5	373	Ustic Haplargids Olney fine sandy loam	A
Sevilleta Grassland	Socorro, NM	Desert grassland	34.34914, −106.88624	16	229	Mesic Ustic Haplocalcids, loamy‐skeletal, carbonatic	A/C
Colorado State University	Fort Collins, CO	Urban Lawn	40.57770, −105.07775	10	408	Fort Collins loam	Ap/Bt
Sky Pond	Rocky Mountain National Park, CO	Alpine meadow	40.27971, −105.66886	1.4	1050	Cryochrepts, Cryumbrepts Rocky sandy loam	O/A/Bw
Loch Vale	Rocky Mountain National Park, CO	Subalpine forest	40.29649, −105.64612	1.4	1050	Cryic spodosols Rocky sandy loam	O/A
Wellrose Farms	Elrosa, MN	Minnesota cornfield	45.60081, −95.01333	5.5	721	Mesic typic Udoll friable silty loam	A
Fenske Lake Cabins	Ely, MN	Coniferous forest	47.99823, −91.91292	4	797	Udic Eveleth‐Eagelsnest‐Conic complex, rocky loam	A/Bw

Note

MAT, MAP, soil classifications, and horizons were determined using the United States Department of Agriculture Natural Resources Conservation Service Soil Resource Reports.

Abbreviations: MAP, mean annual precipitation; MAT, mean, annual temperature.

### 2.2 Soil collection and analyses

All soils were collected from the top ~20 cm of the soil profile. Soils were collected using a 5 cm‐diameter soil auger. The sites Shortgrass Steppe, Limited Irrigation Research Farm, Sevilleta Grassland, Colorado State University, and Wellrose Farms all had either designated plots or sampling areas (e.g., a lawn, a specific cornfield), so within those designated areas 10 soil cores were collected randomly. The sites Sky Pond, Loch Vale, and Fenske Lake Cabins represented broader geographic areas (e.g., a forest), so 10 soil cores were collected at randomly assigned GPS points within ~150 m of the GPS coordinates for the site (Table 1).

Cores were bulked in Ziploc bags, placed on ice in the field to minimize microbial activity, and then refrigerated at 4°C on arrival to the laboratory. Soils collected outside of Colorado were shipped overnight to Colorado State University on ice, sieved to 2 mm and homogenized within 72 h after sampling. Soils collected in Colorado were sieved to 2 mm and homogenized within 24 h after sampling. We chose to sieve soils to break apart soil aggregates to allow for homogenous distribution of the +OC amendment. While this undoubtedly disturbed the soils, it was necessary to adequately address our research question. Because soils could not be assayed immediately after sieving, processed soils were frozen in Ziploc bags at −20°C. This preservation approach both limits microbial activity that could alter soil biogeochemical properties, and therefore allows a more uniform comparison among soils. All incubations and analyses were performed within 2 months of soil collection.

Prior to freezing soils, we measured soil inorganic N (IN) via KCl extractions and calculated soil gravimetric water content (GWC). We also measured soil IN concentrations after all soils had been incubated. Ammonium (NH_4_^+^) and nitrate (NO_3_^−^) were extracted from soils in a 5:1 2 M KCl to soil mixture. Mixtures were mechanically swirled on a shaker table with a 25 mm orbital diameter at 250 rpm for 60 min, settled for 60 minutes, and then gravity filtered. Extracts were analyzed colorimetrically with an Alpkem FIA wet chemistry system (O.I. Analytical). We determined GWC by drying 10 g soil subsamples to a constant weight at 105°C.

Frozen soils were thawed to measure soil pH, soil organic carbon (SOC), and soil organic nitrogen (SON). We measured soil pH from slurries of 10:1 deionized (DI) water to soil with a benchtop meter (Thermo Scientific Orion Star™ A211 Benchtop pH Meter). Frozen soil subsamples were dried in a 60°C oven, ground to powder on a table roller, and then combusted for SOC and SON analysis with a LECO Tru‐Spec CN analyzer (Leco Corp.).

### 2.3 Determination of soil saturation

For all incubations, we held soils at 60% soil saturation. To determine how much water to add to each soil to achieve this saturation, we determined the maximum water holding capacity for each soil. First, we amended thawed subsamples of frozen, field‐moist soil with DI water until fully saturated. Then, we dried these subsamples to a constant weight at 105°C and calculated saturated water content by dividing the g water in the subsample by the subsample dry soil mass. We used this metric rather than % water‐filled pore space because in sieving our soils we broke down all soil pore structures. Finally, to determine the g of water to add to a given soil, we used Equation (1), where g soil corresponds to the g soil incubated, and 0.6 corresponds to the target percent saturation (60%):

(1) $${\text{g H}}_{2}\text{O to add}=\text{g soil}\times \left(\left(\text{SWC}\times 0.6\right)‐\text{soil GWC}\right)$$

### 2.4 Soil incubations

#### 2.4.1 Preparation of OC solutions

We prepared aqueous OC amendments for all treated soils. Equal quantities on a % mass C basis of powdered sodium succinate, sodium acetate, and sodium propionate were dissolved into DI water and diluted serially (two dilutions) to a final concentration of 6.28 mg/L. All organic compounds were obtained from Sigma Aldrich. Aqueous solutions were stored at 4°C when not in use.

The organic acids used are all non‐fermentable, naturally occurring, and have previously been determined by Hedin et al. (1998) to impact denitrifier metabolism. We determined the concentration for the OC amendment by calculating the stoichiometric amount of C required to reduce all the IN in a subsample from the Limited Irrigation Research Farm that was collected prior to our full soil sampling campaign (Table 1). We chose to use that soil for the IN benchmark because we knew this soil would have a high IN concentration, and we wanted to ensure that our OC amendment would provide an ample electron donor supply for microbial metabolism in all the amended soils.

#### 2.4.2 Soil amendments and incubation setup

Soils were separated into either Control, or OC‐amended (+OC) groups. For all incubations, the frozen soil equivalent of 75 g dry soil was weighed into 0.5 L Ball jars and refrigerated overnight to thaw. Prior to treatment, all soils were removed from the refrigerator and warmed to room temperature over ~1 h. To bring all soils to 60% saturation, Control soils were amended with DI water and +OC soils were amended with DI water and 1 ml of the +OC solution. Either amendment was distributed by pipette over the soils. After all liquid was added to a given soil, it was thoroughly mixed to ensure sufficient distribution of OC (if applied) and homogenous saturation.

After all soils were treated, the jars were sealed for incubation. In Stuchiner and von Fischer (2022b) we provide a detailed description of our incubation apparatus, which includes a 1 L Tedlar gas bag attached to the jar headspace to allow for removal of a large quantity of air for subsequent [N_2_O] and isotopic analysis with our laser‐based analyzer (see Section 2.4.3 for details). Prior to incubation, we flushed and filled all incubation apparatuses from a cylinder of custom‐blend air intended to closely emulate Earth's atmosphere (2 ppm CH_4_, 0.450 ppm N_2_O, 21% O_2_, and N_2_ to balance) but without CO_2_ and water vapor, as these gases contribute to optical peak broadening effects (Bowling et al., 2005; Ostrom & Ostrom, 2017; Stuchiner et al., 2020). After incubation vessels were flushed and filled, we used a 3 ml syringe to inject 1 ml of 99 atom percent (AP) ^15^N_2_O into each headspace for isotope pool dilution (details in Section 2.4.4). The syringe was pumped multiple times to ensure that all labeled gas was injected into the incubation vessel.

We performed time zero (*T*_0_) and time 48 (*T*_48_) measurements to enable a 48 h assay duration. Soils were incubated on a lab countertop at room temperature (24°C) for either 60 min (*T*_0_, to allow for homogenization of headspace air following ^15^N_2_O addition) or 49 h (*T*_48_). At the end of the incubation period, we mixed each jar and gas bag's air by attaching a 60 ml syringe to the jar's gas sampling port and, by opening the stopcock valve connecting the jar headspace to the gas bag, then we pumped the syringe for ~ 60 seconds to homogenize the jar headspace and Tedlar gas bag (Stuchiner and von Fischer 2022b). At the time of sampling, we connected our incubation apparatus directly to the gas analyzer‐scrubber system (see Section 2.4.3 for details). As the analyzer removed air from the jar headspace, air was simultaneously removed from gas bag, thus keeping the jar air pressure at atmospheric pressure.

#### 2.4.3 Measurements of N_2_O and CO_2_ concentration, and of N_2_O isotopic composition

After 60 min (*T*_0_) or 49 h (*T*_48_), incubation vessels were analyzed for N_2_O concentration and isotopic composition by a laser‐based LGR N_2_O isotopic analyzer (Los Gatos Research N_2_O Isotopic Analyzer model 914‐0027; ABB‐Los Gatos Research). Gas was sampled from each incubation vessel for 12–15 min (or until the N_2_O concentration stabilized) at a flow rate of 42 ml/min into the analyzer. We removed CO_2_, water vapor and volatile organics from the sample air using a Nafion–carbosorb–silica gel scrubbing system. Removing these gases minimizes the optical peak‐broadening effects inherent to laser‐based analyzers (Stuchiner et al., 2020).

All raw concentration data for each N_2_O sample were exported to Excel (version 16.52), where they were trimmed to only include stabilized N_2_O isotopocule readings (the final ~5 min of sampling). These values were used to calculate average N_2_O and isotopomer concentrations. All reported concentrations were then corrected against calibrations using the model and approach described in Stuchiner et al. (2020).

We measured accumulated CO_2_ in the *T*_48_ incubation jars to characterize microbial respiration. Air samples of 3 ml were drawn with a 3 ml syringe from the remaining headspace air in the incubation jars at the end of the *T*_48_ N_2_O sampling. Samples were analyzed on a laser‐based LGR Greenhouse Gas Analyzer (Los Gatos Research Greenhouse Gas Analyzer model 908‐0007‐001; ABB‐Los Gatos Research). Sample air was admitted to the analyzer by injecting 3 ml of air samples into a continuous flow of zero‐grade air (80:20 N_2_:O_2_ blend; Airgas Industries) that was connected to the analyzer via an open split. All baseline GHG concentrations were <10 ppm, and each injected sample yielded a CO_2_ concentration peak. The height (i.e., maximum concentration) of these peaks were calibrated against a four‐point calibration curve generated with CO_2_ standards from Airgas (1023, 5000, 10,000, 60,000 ppm CO_2_) that were also injected into the analyzer when zero‐grade air was flowing through it.

#### 2.4.4 Flux calculations and isotope pool dilution

We calculated soil net N_2_O emissions using measures of the headspace N_2_O concentration and calculated gross N_2_O consumption using isotope pool dilution (Kirkham & Bartholomew, 1954; von Fischer & Hedin, 2002). Net N_2_O flux was calculated from the difference in N_2_O concentration between *T*_0_ and *T*_48_ and is presented in units ng N_2_O‐N/g dry soil/day.

Gross N_2_O consumption was quantified based on the disappearance of ^15^N‐labeled N_2_O. We followed the convention of von Fischer and Hedin (2002), which considers N_2_O production rates to be relatively constant over the measurement interval, but N_2_O consumption to be a first‐order function of N_2_O concentration. Thus, we adopted their Equation ((1), (4)) and modified it as:

$$F=\frac{d\left[N_{2}O\right]}{\mathrm{dt}}=P‐k\left[N_{2}O\right].$$

where *F* is the net flux rate, [N_2_O] is the N_2_O concentration in the jar headspace, *P* is the production rate, and *k* is the first‐order consumption rate constant. Note that the gross consumption rate is the product of *k* and the change in N_2_O concentration. The focus in our study was to quantify the N_2_O consumption rate, which is essentially measuring the uptake constant, *k*. The amount of labeled N_2_O falls exponentially over time following Equation (2),

(2) $${\text{*N}}_{2}O\left(t\right)={\text{*N}}_{2}O_{0}e^{‐kt}$$

which is equivalent to Equation (3),

(3) $${\text{ln*N}}_{2}O\left(t\right)={\text{ln*N}}_{2}O_{0}‐k\cdot t$$

where *N_2_O(*t*) is the amount of labeled N_2_O at time *t*. Substituting *N_2_O_48_ and *t* = 48, the solution for *k* is:

(4) $$k=\frac{{\text{ln*N}}_{2}O_{0}‐{\text{ln*N}}_{2}O_{48}}{48}$$

We calculated *N_2_O, the amount of labeled N_2_O in the incubation jar headspace, as the product of the total headspace N_2_O concentration and the AP excess (APE) ^15^N^bulk^ in the N_2_O.

Determination of the APE follows a series of calculations. First, after N_2_O and its isotopomers were calibrated using equations from Stuchiner et al. (2020), we determined ^15^N^bulk^ by calculating the average concentration of ^15^N^α^ and ^15^N^β^ using Equation (5):

(5) $${}^{15}N^{\text{bulk}}=\frac{\left[{}^{14}N^{15}N{}^{16}O\right]+\left[{}^{15}N{}^{14}N{}^{16}O\right]}{2}$$

Next, we subtracted ^15^N^bulk^ from the total N_2_O concentration for a given sample to calculate the light (^14^N^14^N^16^O) fraction of emitted N_2_O. Then we calculated the AP of the heavy isotope (AP) for each sample using Equation (6)

(6) $$\text{AP}=\frac{{}^{15}N^{\text{bulk}}}{{}^{15}N^{\text{bulk}}+{}^{14}N{}^{14}N^{16}O}$$

Then, we subtracted the AP of natural abundance ^15^N_2_O from the observed AP to obtain the APE of ^15^N_2_O present in the incubation headspace. We assumed a natural abundance δ^15^N of N_2_O to be −35‰, which is equivalent to AP of 0.3535% (Hu et al., 2015).

These excess ^15^N_2_O concentrations were used in Equation (4) to calculate *k*, the first order rate constant for N_2_O consumption. Gross N_2_O consumption rates were then calculated by multiplying each k value by 0.332, the mean atmospheric N_2_O concentration in parts per million (ppm) in 2019 (www.N2Olevels.org). Each gross consumption rate was expressed in units ng N_2_O‐N/g dry soil/day.

#### 2.4.5 Leak test of incubation apparatus

A separate test assessed the gastight seals of the incubation vessels. Twelve incubation vessels were flushed and filled with zero‐grade air and injected with 1 ml of 99 AP ^15^N^15^N^16^O into the headspace of each jar using a 3 ml syringe. After mixing the jar and gas bag air thoroughly, this raised the N_2_O concentration and the δ^15^N^bulk^ on average to ~500 ppb and ~6300‰, respectively. Six vessels were sampled for N_2_O and isotopomer concentrations at T_0_ and the remaining six vessels were sampled at *T*_48_. All samples were measured on our LGR N_2_O isotopic analyzer. *t*‐Tests revealed that changes from *T*_0_ to *T*_48_ in total N_2_O concentration were not significant, while changes in concentration of enriched ^15^N_2_O was less than 2.3% for δ^15^N^α^ and δ^15^N^β^.

#### 2.4.6 Post‐incubation soil and genetic measurements

After all gas had been sampled for the *T*_48_ incubations, soil replicates from each group were bulked into a Ziploc bag and re‐homogenized. Soil IN was measured immediately thereafter, as in Section 2.2. Two‐gram subsamples from each treatment group were frozen at −80°C in preparation for microbial genetic analysis, and remaining soils were frozen at −20°C.

To measure the abundances of key N‐cycling genes (nifH, nirK, clade I nosZ), we extracted DNA from soils that had been frozen at −80°C using a Qiagen Powersoil Pro kit according to the manufacturer's instructions (Qiagen). The nifH gene encodes the enzyme that catalyzes N‐fixation, the nirK gene encodes the enzyme that catalyzes reduction of nitrite (NO_2_^−^) to nitric oxide (NO) and is, thus, characteristic of incomplete denitrification, and the nosZ gene encodes the enzyme that catalyzes reduction of N_2_O to N_2_ and is thus characteristic of complete denitrification (Domeignoz‐Horta et al., 2015; Kuypers et al., 2018; Sanford et al., 2012). We acknowledge that nirS is also measured to represent incomplete denitrification (Sanford et al., 2012), however cost constraints prevented us from quantifying that gene. The quality (A260/A280) and concentration from each DNA sample was determined spectrophotometrically using a NanoDrop instrument (Thermo Scientific). All samples were diluted to 10 ng/μl and then we characterized the abundances of the abovementioned N‐cycling genes using qPCR analysis. All qPCR reactions were performed in 96‐well plates using an ABI Prism 7500 Sequence detection system (Applied Biosystems). The following thermal cycling program was used for all genes: 95°C for 15 s, 63–58°C for 30 s (−1°C by cycle), 72°C for 30 s, 80°C for 15 s, six cycles, as in Trivedi et al. (2012). For each soil sample, qPCR amplification was performed for each N‐cycling gene four times. Gene abundances (e.g., gene copy numbers) were determined by using simple linear regressions that related the cycle threshold (Ct) value for each sample to known Ct values from a standard curve. Standard curves for each gene were generated by preparing an 8‐fold serial dilution of plasmids that contained the gene of interest.

We acknowledge that clade II nosZ has been identified as an important gene in the N_2_O → N_2_ reduction step in the denitrification pathway (Almaraz et al., 2020; Chee‐Sanford et al., 2020; Jones et al., 2014). However, due to financial constraints and a lack of primers we could not quantify clade II nosZ gene abundances in this study.

### 2.5 Data analyses

#### 2.5.1 Calculation of OC:Control ratios

To determine the effect of the +OC treatment, we compared +OC versus Control treatments for net N_2_O emissions, gross N_2_O consumption, soil NO_3_^−^ and NH_4_^+^ concentrations, microbially emitted [CO_2_], and the gene abundances of nifH, nirK, and clade I nosZ for all soils at the conclusion of incubations. To quantify the effect of the OC amendment, we calculated response ratios as the +OC:Control ratio for each variable measured. Because the +OC and Control replicates for a given soil were not paired, we calculated the mean for the Control for each property, and then we divided each +OC value by the mean Control value. Thus, for a ratio >1 the +OC treatment increased that property, while for a ratio <1 the +OC treatment decreased that property. When applicable, +OC:Control response ratios were used in all statistical analyses.

#### 2.5.2 Statistical analyses

All raw data were collected and collated in Excel, and statistical analyses were performed in RStudio (version 4.0.2 (2020‐06‐22)—“Taking Off Again” © 2020 The R Foundation for Statistical Computing). All data used for statistical analyses are openly available at Mountain Scholar: Digital Collection of Colorado and Wyoming (Stuchiner & von Fischer, 2022a). Differences among initial soil NO_3_^–^ and NH_4_^+^ concentrations were determined using one‐way ANOVAs. Associations among all inherent soil properties and +OC:Control ratios and ICDE were examined using a MANOVA (Box 1), with subsequent one‐way ANOVAs performed to assess differences among all soils for each property individually. Residuals were examined for departure from normality. All N_2_O production and consumption data from all soil incubations were log‐transformed to meet assumptions of normality in residuals.

We also performed a principal component analysis (PCA) to examine the associations among variables and ICDE. We used the package factoextra to visualize the results of the PCA and we used the package missMDA to impute the data set. To determine if there were significant associations among properties and ICDE we performed *t*‐tests comparing the Yes ICDE versus No ICDE coordinate loadings for PCs 1–4.

## 3 RESULTS

### 3.1 N_2_O emission and consumption rates

**FIGURE 1:**
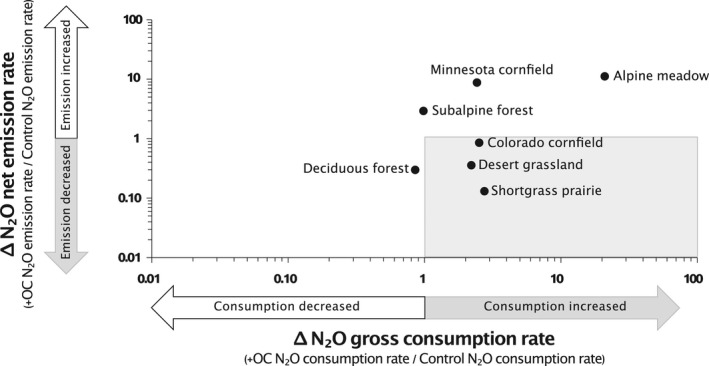
Change in (or “Δ”) net N_2_O emission versus gross N_2_O consumption rates for all soils excluding the Urban Lawn soils, in which no N_2_O consumption was observed following +OC amendment. Δ values were calculated as +OC:Control response ratios for gross consumption rate and net emission rate (both rates are in ng N_2_O‐N/g dry soil/day). Soils included in the grey box had **I**ncreased N_2_O **C**onsumption and **D**ecreased N_2_O **E**missions (ICDE). For net N_2_O emission, *n* = 6 in all cases, except the alpine meadow, in which *n* = 12. For gross N_2_O consumption, *n* = 12 for the alpine meadow, *n* = 5 for the Minnesota cornfield, *n* = 2 for the coniferous forest, and in all other cases *n* = 6. Because the urban lawn had no measurable N_2_O consumption in the +OC treatment, it could not be plotted on this figure; it had a 17‐fold increase in N_2_O emission rate. OC, organic carbon

Organic carbon additions stimulated gross N_2_O consumption in six of the eight incubated soils (Figure 1). This includes the alpine meadow, Colorado cornfield, desert grassland, Minnesota cornfield, shortgrass prairie, and subalpine forest. However, soils differed in whether +OC led to an increase versus decrease in net N_2_O emissions. The alpine meadow, Minnesota cornfield, and subalpine forest soils all had increased N_2_O emissions in +OC compared with Control soils, whereas the Colorado cornfield, desert grassland, and shortgrass prairie soils all had decreased N_2_O emissions in +OC compared with Control soils (rates of emission and consumption presented in Figure S1). Consequently, these latter three soils showed ICDE (Box 1). Only the coniferous forest decreased in both net emissions and gross consumption following OC amendment. Note that the urban lawn soil is not included in Figure 1 because no gross consumption was observed; only the urban lawn had increased net emissions and no gross consumption following OC amendment (data not shown). On average, among the seven soils plotted in Figure 1, there was a 366% increase in N_2_O consumption following OC amendment, and a 250% increase in N_2_O emissions following OC amendment.

### 3.2 Differences among soil properties and microbial gene abundances

**FIGURE 2:**
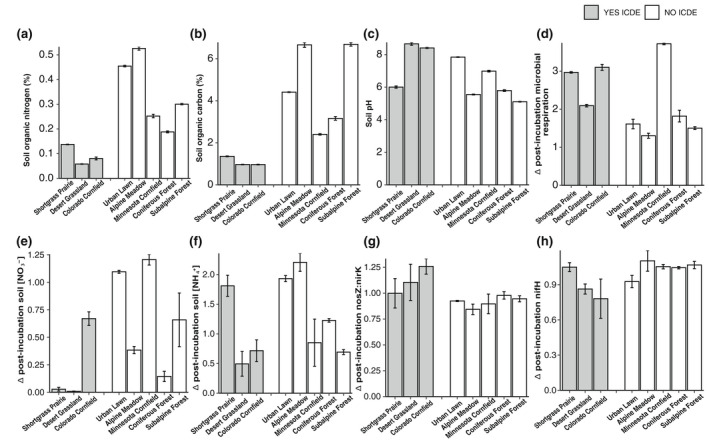
Panels (a–h) show the soil properties or microbial genes abundances among all soils tested, with panels in order from most (a) to least (h) significance in one‐way ANOVA for soils. Panels (a–c) are direct measures of soil properties that were measured prior to the incubation experiments, whereas panels (d–h) are all Δ values for +OC:Control soil properties and N‐cycling gene abundances. The Δ nosZ:nirK (g) is a ratio‐of‐ratios, in which the nosZ:nirK ratio for +OC soils is divided by the nosZ:nirK ratio for the Control soils. We partitioned soils by Yes ICDE (grey bars) versus No ICDE (white bars) groupings to highlight properties that Yes ICDE had in common and how collectively Yes ICDE versus No ICDE differed in their properties. Error bars are ±1 SE from the mean. Please see Table S1 for *n*‐values and units for each property

We performed a MANOVA regressing all the soil and microbial properties we measured versus the ICDE categories (hereafter categorized as “Yes ICDE” or “No ICDE”). The MANOVA revealed that at least one of the properties impacted whether a soil showed ICDE or not (*p* < .0001). To better discern which properties were important drivers of ICDE, we performed subsequent one‐way ANOVAs to assess differences among soils for each property individually. As illustrated in Figure 2, all properties were highly significantly different among soils (*p* < .0001, Figure 2a–f) except for the microbial gene abundances (*p* = .095 for Δ nosZ:nirK and *p* = .148 for Δ nifH, Figure 2g,h, respectively).

**FIGURE 3:**
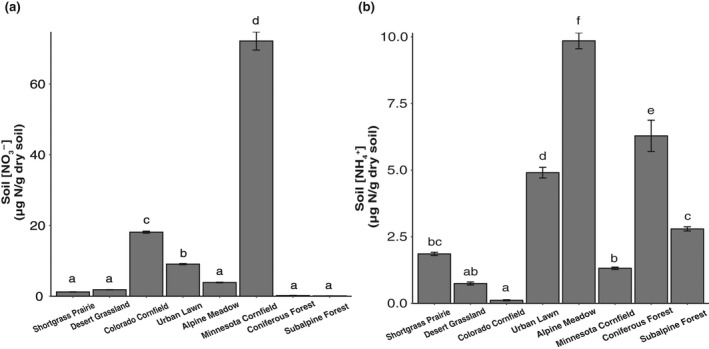
Initial NO_3_^−^ (a) and NH_4_^+^ (b) concentrations in soils prior to incubations. Soil extractable NO_3_^−^ and NH_4_^+^ were measured within 24–72 h of field sampling. Letters correspond to significantly different mean concentrations. In all cases, *n* = 4, and error bars are ±1 SE from the mean

ANOVAs indicated that soils had significant differences in IN pools prior to incubation. There was a significant difference among all soils for NH_4_^+^ (*p* < .0001) and NO_3_^−^ (*p* < .0001) concentrations (Figure 3). Tukey pairwise comparisons revealed that there were multiple significant differences among soil NH_4_^+^ concentrations pre‐incubation, whereas there were fewer differences among soil NO_3_^−^ concentrations pre‐incubation (Figure 3). However, the differences among NO_3_^−^ concentrations were more pronounced, with the Minnesota cornfield having the highest soil NO_3_^−^ concentration (*p* < .0001 in all cases), the Colorado cornfield having the second highest soil NO_3_^−^ concentration (*p* < .001 in all cases), and the urban lawn having the third highest soil NO_3_^−^ concentration (*p* < .001 for all comparisons, except *p* = .0001 for urban lawn vs. desert grassland and *p* = .0076 for urban lawn vs. alpine meadow, Figure 3).

### 3.3 Associations between predictor variables and ICDE

**FIGURE 4:**
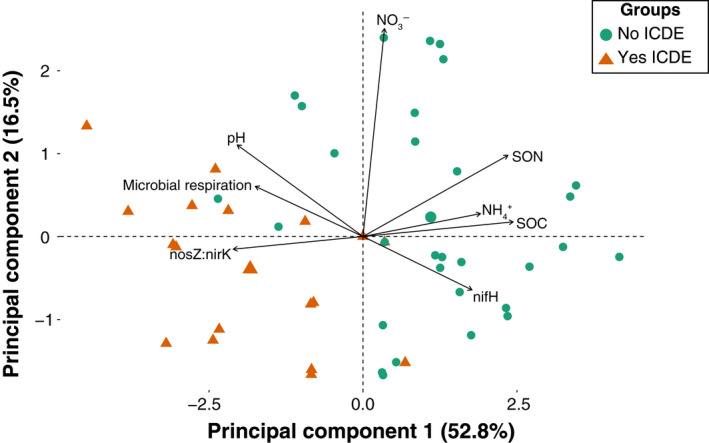
Loadings and biplot for principal component analysis. Orange triangles correspond to Yes ICDE soils, whereas green circles correspond to No ICDE soils. See Table S1 for *n*‐values for each variable

The PCA grouped variables to reveal associations among predictor variables and ICDE. Principal component (PC) 1 explained more than 50% of the variation among the variables, whereas PC2 explained approximately 17% of the variation among the variables (Figure 4; see Figure S2 for grouping of PCA responses by soil). Although PC1 accounted for the most associations among variables and ICDE, we ranked the loadings associated to each predictor variable for PCs 1–4. These loadings and rankings are summarized in Table 2.

**TABLE 2:** Predictor variables, their associated p‐values from individual ANOVAs, and loadings for each variable for principal components (PCs) 1–4

Predictor	*p*‐value (from ANOVAs)	PC1: rank (loading)	PC2: rank (loading)	PC3: rank (loading)	PC4: rank (loading)
Significant association with Yes/No ICDE?	NA	**Yes** *p* < .0001	**No** *p* = .07	**No** *p* = .74	**No** *p* = .09
SON	<.0001	2 (0.43)	3 (0.32)	6 (−0.20)	6 (−0.08)
SOC	<.0001	1 (0.44)	7 (0.06)	5 (−0.21)	2 (0.35)
pH	<.0001	4 (−0.37)	2 (0.36)	4 (−0.25)	4 (−0.24)
Δ Microbial respiration	<.0001	7 (−0.32)	5 (0.20)	1 (0.63)	5 (−0.17)
Δ Soil NO_3_^−^	<.0001	8 (0.06)	1 (0.82)	7 (0.18)	3 (0.25)
Δ Soil NH_4_^+^	<.0001	5 (0.35)	6 (0.09)	8 (−0.05)	1 (−0.84)
Δ nosZ:nirK	.095	3 (−0.38)	8 (−0.05)	3 (−0.30)	7 (−0.03)
Δ nifH	.148	**6 (0.32)**	4 (−0.21)	2 (0.57)	8 (0.01)

Note

Each loading has a corresponding rank associated with it, and ranks are ordered by the loading absolute values, or magnitude of loading. Ranks are listed next to their loading value in parentheses, with the top four ranks of each PC in bold font. Variables are listed in the table in order of significant differences among soils, from most significant difference to least significant difference. Significant associations with ICDE for each ranking were calculated using two‐sample *t*‐tests (Figure S3). The first three predictors were all direct measures of soil properties that were measured prior to the incubation experiments. The last five predictors are all Δ values for +OC:Control soil properties and N‐cycling gene abundances.

Abbreviations: SOC, soil organic carbon; SON, soil organic nitrogen.

Furthermore, *t*‐tests comparing Yes versus No ICDE coordinates for the different soils illustrated there was a significant association with ICDE for PC1, but not for PC2 (Table 2). However, the association with ICDE and PC2 was borderline significant (*p* = .07). Plots associated with *t*‐tests can be found in the Supplementary Information (Figure S3). From PC1, the loadings indicate that Yes ICDE soils associated most strongly with low SOC, low SON, high Δ nosZ:nirK, and high soil pH (Table 2). The Yes ICDE soils also associated with low Δ soil NH_4_^+^, low Δ nifH abundance, and high Δ microbial respiration on PC1, but associated very weakly with Δ soil NO_3_^−^ (Table 2). However, Δ soil NO_3_^−^ was the most strongly loaded variable on PC2, and the Yes ICDE soils associated most strongly with low Δ soil NO_3_^−^ (Figure 4, Table 2).

## 4 DISCUSSION

We sought to stimulate N_2_O consumption in diverse soils by adding OC to manipulate the N_2_O reducing potential of these soils. We hypothesized that providing excess electron donors to soil microbes would stimulate N_2_O → N_2_ reduction by inducing denitrifiers to use N_2_O as a terminal electron acceptor (Firestone & Davidson, 1989; Hedin et al., 1998; Ostrom et al., 2002). Our hypothesis was supported, in that amending soils with OC stimulated N_2_O consumption in most of the soils tested (six out of eight soils). However, the joint response of consumption and N_2_O emission differed among soils. Three soils showed ICDE while three other soils increased N_2_O consumption but also increased N_2_O emissions (Figure 1). Additionally, one soil decreased both consumption and emissions, and one soil only increased emissions (Figure 1). Our data on various soil and microbial genetic properties provide some insights into the dynamics of N_2_O metabolism that we observed. With an improved understanding, we hope this and future studies following this approach will enable better prediction of a soil's potential for N_2_O consumption, based on its properties and microbial gene abundances. Net uptake of N_2_O from the atmosphere has been documented in gas flux chamber studies for decades (Almaraz et al., 2020; Chapius‐Lardy et al., 2007; Schlesinger, 2013), but only recently has N_2_O consumption been recognized as a force that regulates net N_2_O emissions (Shan et al., 2021). Our findings likely have implications for explaining these field‐scale observations.

Our interpretation of ICDE rests heavily on the concept of soil redox dynamics, both at the bulk soil scale and across soil anoxic microsites. From a bulk redox perspective, the energetic metabolism of soil microbes requires both electron donors (usually some form of OC) and electron acceptors, such as O_2_, NO_3_^−^, MnO_2_, Fe^2+^, SO_4_^2−^, CO_2_, etc. These electron acceptors exist on a “thermodynamic ladder” that ranks their energetic favorability from greatest to least, with the oxidation of O_2_ being the most energetically favorable and the oxidation of CO_2_ being the least energetically favorable (Hedin et al., 1998). A given soil can range from being electron donor limited (typically oxic environments) to electron acceptor limited (typically anoxic environments). Interestingly, N_2_O reduction falls between NO_3_^−^ and MnO_2_ reduction on the redox ladder.

Because of this sequencing, we originally hypothesized that further additions of OC (e.g., electron donors) would both drive O_2_ consumption, creating a greater soil volume where N_2_O reduction could occur (Sihi et al., 2020; Wu et al., 2013), and also stimulate the metabolism of denitrifiers directly by adding substrates known to be used by those bacteria. Decreased N_2_O emissions in response to OC amendment have been observed in previous studies. For example, Cavigelli and Robertson (2001) found that the N_2_O:N_2_ production ratio for pure cultures of denitrifying bacteria fell as the supply ratio of OC:NO_3_^−^ became greater. Likewise, along a redox gradient at a soil‐stream interface, Hedin et al. (1998) saw C additions lead to lower N_2_O emissions. However, we now hypothesize that adding OC to soils might not increase N_2_O consumption *unless* the added electron donors expand anoxic regions in the soil where N_2_O consumption is thermodynamically favorable. We predict that such an enhancement of N_2_O consumption will depend on the physical and biogeochemical features of the soil such as aggregation, pore structure, and micro‐scale hotspots of respiration.

### 4.1 N_2_O consumption rate increased in response to +OC treatment

Gross N_2_O consumption rates increased in response to +OC treatment in most, but not all, the soils tested (Figure 1). Differences in redox potential among the soils may have contributed to some of the differential emission and consumption behaviors we observed (Cheng et al., 2017; Senbayram et al., 2012; Wang, Chen, et al., 2021; Włodarczyk et al., 2021). One possible explanation is that the soils that showed ICDE became sufficiently reducing in response to OC amendment. If the OC amendment enabled electron donor supply to exceed electron acceptor NO_3_^−^ supply, the soil microbes would have the redox capacity to fully deplete the NO_3_^−^ pool for energy metabolism (Taylor & Townsend, 2010). As a result, microbes would need to use N_2_O as an alternative electron acceptor, which would stimulate complete denitrification and consequently, ICDE. Previous work also supports this explanation. Under specific OC amendments, soil NO_3_^−^ concentration and N_2_O emissions have been shown to decrease, indicating more complete denitrification (Dodla et al., 2008; Gillam et al., 2008; Hill et al., 2000; Lan et al., 2017; Senbayram et al., 2012; Wang, Chen, et al., 2021). Furthermore, the differential balance of electron donors and acceptors among the soils we tested is likely regulated by other features of the soils (Gu & Riley, 2010; Jamali et al., 2016; Neubauer et al., 2005; Sutton‐Grier et al., 2011). The soils that showed ICDE differed notably from the soils that did not in specific properties (Figures 2 and 4). We argue in the next section that these properties contribute to why soils did or did not show ICDE.

### 4.2 Assessing soil properties to predict ICDE

From a management perspective, it would be ideal to predict which soils are more likely to respond to +OC treatment with ICDE. In general, the soils with ICDE had markedly lower SOM, and in response to OC amendment, showed greater depletion of soil NO_3_^−^, larger increases in soil microbial respiration, and bigger increases in the ratio of nosZ:nirK genes (Figure 2). These findings support our initial hypothesis that N_2_O consumption is limited by reductants, and it supports our follow‐up hypothesis, that for C additions to induce ICDE, the additions need to expand the N_2_O reducing regions in the soil without over‐stimulating N_2_O production via denitrification.

Soils that showed ICDE were OM‐limited, and the OC amendment induced heightened microbial activity, and altered the soil redox environment in a way that both drove net NO_3_^−^ consumption and increased gross N_2_O consumption (Figures 1 and 2). This notion is supported by the PCA, which heavily weighted SOC, SON, microbial respiration, and nosZ:nirK with the soils that showed ICDE along PC1 (Figure 4), and other studies (Buchen et al., 2019; Gallarotti et al., 2021; Guo et al., 2020; Miller et al., 2009; Voigt et al., 2020). The significant role of pH in affecting N_2_O consumption, as revealed in PC1 and the pH one‐way ANOVA, is consistent with the effect of high pH being more favorable for nosZ enzyme activity (Figures 2 and 4; Blum et al., 2018; Richardson et al., 2009).

Soils that did not show ICDE either had stimulation of N_2_O production that exceeded the N_2_O consumption change (alpine meadow, Minnesota cornfield, subalpine forest), or they had depressed N_2_O consumption following OC amendment (coniferous forest, urban lawn). The soils that did not show ICDE were characterized by high SOM, and in response to OC amendment, had variable changes in soil NO_3_^−^ and in soil microbial respiration, and little change in the ratio of nosZ:nirK genes (Figures 2 and 4; Figure S2). Although some non‐ICDE soils had higher initial soil NH_4_^+^ and lower initial soil NO_3_^−^ concentrations (Figure 3), these antecedent conditions did not effectively predict redox‐reducing potential (Balser & Firestone, 2005; Cardenas et al., 2019; Zhang et al., 2021). Unlike soils with ICDE, which were clustered together in the PCA (Figure 4), the non‐ICDE soils were widely scattered in PC1 and PC2 (Figure S2), suggesting that perhaps disparate biogeochemical drivers can lead to a non‐ICDE response.

For the soils with both increased N_2_O consumption and emissions (alpine meadow, Minnesota cornfield, subalpine forest), it is possible that the OC additions helped to create suboxic/anoxic microsites by stimulating microbial respiration (Figure 2d). These microsites could have hosted more denitrification, both incomplete and complete (e.g., both increased N_2_O emissions and consumption). The increased NO_3_^−^ production in the Minnesota cornfield also aligns with the idea of coupled nitrification‐denitrification, which has been associated with anoxic microsites in oxic soils (Kremen et al., 2005; Stevenson et al., 2011; Surey et al., 2020; Wu et al., 2018). Additionally, the higher SOM in these soils may have permitted less efficient energy metabolism, which can result in more denitrification overall (Scheer et al., 2020; Senbayram et al., 2012; Surey et al., 2020). This may explain why these soils had increased N_2_O consumption *and* increased N_2_O emissions.

The N_2_O consumption response was weak in some soils (Figure 1). High NO_3_^−^ concentrations have been shown to suppress clade I nosZ transcription in denitrifiers (Hallin et al., 2018). This suppression of denitrification following suppressed clade I nosZ transcription could explain why the Minnesota cornfield and urban lawn soils had both weak N_2_O consumption responses and increased NO_3_^−^ concentrations following OC amendment (Figures 1 and 2e). The N_2_O consumption response of the coniferous forest soil was the most suppressed of all soils (Figure 1). This soil was among the lowest for both respiration response to OC amendment and initial NO_3_^−^ concentrations, suggesting low denitrification potential (Figures 2d and 3a).

Our results suggest that land managers can reduce N_2_O emissions from low OC soils using OC additions. We hypothesize that OC additions to low OC soils will relax C‐limitation, promote soil anoxia, and, in turn, stimulate the reduction of both NO_3_^−^ and N_2_O via denitrification. We expect that future work can build on this hypothesis to identify the biogeochemical conditions and dynamics where N_2_O consumption can be enhanced for GHG management.

### 4.3 Carbon limitation threshold to ICDE

**FIGURE 5:**
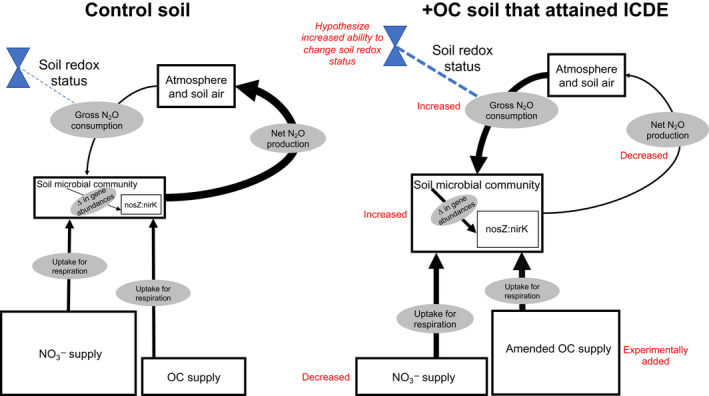
Conceptual diagram compare‐contrasting a Control soil to an OC amended counterpart that showed ICDE. Boxes correspond to pools, arrows correspond to fluxes, and ovals describe the fluxes. Blue valves correspond to the ability for a soil to change its redox status (mediated by aggregation, pore structure, micro‐scale hotspots of respiration, etc.). Red text next to pools, fluxes, or valves provide details for how they change in response to +OC treatment or provide a hypothesized change. We hypothesize soils that showed ICDE overcame an OC limitation and were freed from electron donor limitations to increase microbial activity and subsequently change the soil redox environment. These changes contributed to increased transcription of nosZ relative to nirK, thus driving an increase in gross N_2_O consumption. The blue dotted line illustrates the capacity in the soil to enable N_2_O consumption, and the thickness of the line indicates if the capacity is high or low. We posit the soils that showed ICDE had high capacity to expand regions of anoxia where N_2_O consumption was thermodynamically favorable. The sizes of pools and fluxes are not to scale, but rather illustrate general changes in pool sizes or process rates. OC, organic carbon

From our findings, we present a conceptual framework to explain how +OC treatment stimulated ICDE (Figure 5). Broadly, to stimulate gross N_2_O consumption, soil microbes must surpass a C‐limitation threshold to induce sufficient anoxia for N_2_O consumption to occur. We posit this is how SOC‐poor soils showed ICDE in our soil incubations. Conversely, the other soils that did not show ICDE had higher SOC and might not have been (as) reductant limited. As a result, those soils might have been less responsive to the OC amendment (Figures 2 and 4).

How might the soils that did not show ICDE respond to an even larger OC amendment? We hypothesize that the electron donor supply must create sufficiently reducing conditions to show ICDE. If supported, this suggests that the soils that did not show ICDE were still reductant (e.g., OC) limited. This raises the question, where does the OC‐limitation threshold lie for different soils, and how can we predict it? What other variables mediate this threshold? Being able to identify the amount of OC a soil requires to induce ICDE, and if in fact that soil is a good candidate for +OC treatment (e.g., what is its aggregation potential?) could be useful for screening soils as targets for N_2_O consumption management.

### 4.4 Future work

It remains unresolved how the +OC treatment impacts other soil microbial community responses. Specifically, we did not examine how amending soils with OC affected CH_4_ emissions, or if CO_2_ emissions from microbial respiration offset N_2_O consumption (Li et al., 2005; Zaehle et al., 2011; Zhou et al., 2017). While we expect that amending soils with our +OC treatment would stimulate microbial respiration, it would be ideal if the +OC treatment also stimulated an ICDE‐induced net N_2_O reduction to offset any CO_2_ or CH_4_ emissions from +OC treatment. Additionally, as the N_2_O management community continues to explore organic amendments to decrease net N_2_O emissions, it will be important to better understand which specific additions are most effective at reducing net emissions. Studies have illustrated that certain OC amendments can increase net N_2_O emissions (Guenet et al., 2021). To use these amendments successfully, it will be crucial to better understand which organic amendments to use or avoid on different soils, as it is possible amendments may differ in their efficacy by soil type (Guenet et al., 2021). Managing N_2_O consumption could be central for GHG management, particularly in agroecosystems. These systems are projected to become an increasingly important source of GHGs as the human population grows, so developing strategies to curtail their emissions while sustainably feeding people will be instrumental for the future (Bakken & Frostegård, 2020; Battye et al., 2017; Kanter, Del Grosso, et al., 2020).

## CONFLICT OF INTEREST

The authors declare no conflict of interest.

## Supporting information

Supplementary Material

## Data Availability

The data that support the findings of this study are openly available Mountain Scholar: Digital Collections of Colorado and Wyoming at http://dx.doi.org/10.25675/10217/234597.
