# Supplementary material for: Using isotope pool dilution to understand how organic carbon additions affect N2O consumption in diverse soils
Source: Glob Chang Biol. 2022 May 4;28(13):4163–79. doi: 10.1111/gcb.16190 (PMC9321687; doi:10.1111/gcb.16190)
Supplement: Supplementary file 1 — Supplementary Material [file GCB-28-4163-s001.docx]

*Global Change Biology*

Supporting Information for

**Using isotope pool dilution to understand how organic carbon additions affect N_2_O consumption in diverse soils**

E.R. Stuchiner^1,2^ and J.C. von Fischer^1,2^

^1^Graduate Degree Program in Ecology, Colorado State University, Fort Collins, CO

^2^Department of Biology, Colorado State University, Fort Collins, CO

**Contents of this file**

Table S1

Figure S1

Figure S2

Figure S3

**Table 1.** Numbers of replicates (n) for all soil and microbial genetic properties measured before or after soil incubations (all properties from Figure 2 in the paper). Unless otherwise stated, n-values are the same for a given property across all soils tested. The n-values for all properties measured before incubations correspond to technical replicates from each bulked and homogenized soil. The n-values for all properties measured after incubation correspond to technical replicates from bulked and homogenized soils from incubation jars from the same treatment. For example, the contents from all the +OC Shortgrass prairie jars would be combined into one Ziploc bag and homogenized, whereas the contents from all the Control Shortgrass prairie jars would be combined into one Ziploc bag and homogenized, and so on for all the other soils tested. *Note: soil NO_3_^-^ and NH_4_^+^ concentrations are measured before and after incubations. The before-incubations n-values are included in Figure 3 in the main text of this paper, whereas the after-incubations n-values are included here.*

| **Property measured** | **Units of measured property** | **n-value** | **Measured before or after soil incubations?** |
| --- | --- | --- | --- |
| Soil organic carbon (SOC) | Percent, % | 5, except n = 4 for:  - Minnesota cornfield  - Coniferous forest | Before |
| Soil organic nitrogen (SON) | Percent, % | 5, except n = 4 for:  - Minnesota cornfield  - Coniferous forest | Before |
| Soil pH | Percent H^+^ | 5 | Before |
| Microbial respiration | µg CO₂-C * g soil^-1^ day^-1^ | 6 | After |
| Soil [NO_3_^-^] | µg N * g dry soil^-1^ day^-1^ | 3 | After |
| Soil [NH_4_^+^] | µg N * g dry soil^-1^ day^-1^ | 3 | After |
| nosZ:nirK | Ratio (of log_10_ gene copy numbers) | 3, except n = 4 for:  -Colorado cornfield  and n = 2 for:  -Subalpine forest | After |
| nifH | Log_10_ gene copy number | 3, except n = 4 for:  - +OC Colorado cornfield  - Control Coniferous forest  - Control Alpine meadow  - +OC Urban Lawn  - Control Urban Lawn | After |

|  |  |
| --- | --- |

**Figure S1.** N_2_O emission and consumption rates for Control and +OC-treated soils. For the soils that showed ICDE (shortgrass prairie, desert grassland, Colorado cornfield), note that net N_2_O emissions decrease and gross N_2_O consumption increases following OC-amendment. For net N_2_O emission, n = 6 in all cases except the alpine meadow, in which n = 12. For gross N_2_O consumption, n = 12 for the alpine meadow, n = 5 for the Minnesota cornfield, n = 2 for the coniferous forest, and in all other cases n = 6. Error bars are ± one SE from the mean.

**Figure S2.** Loading and biplot for PCA. All yellow asterisks correspond to Yes ICDE soils, whereas all other shapes correspond to No ICDE soils. There is clustering (e.g., coherency) in associations among the principal components for the Yes ICDE soils, whereas the No ICDE soils, which clustered by site, do not associate with each other. See table S1 for n-values for each variable.

|  |  |
| --- | --- |
|  |  |

**Figure S3.** T-tests comparing Yes vs. No ICDE mean coordinate loadings among the properties used in the Principal Components Analysis (PCA) in this study. Only PC1 yielded significant differences in mean loading for Yes vs. No ICDE (p < 0.0001), however PC2 yielded borderline significant differences in mean loading for Yes vs. No ICDE (p = 0.07). In all cases n = 18 for Yes ICDE coordinates, and n = 30 for No ICDE coordinates. Error bars are ± one SE from the mean.
